# A Quick Surgical Treatment of Conjunctivochalasis Using Radiofrequencies

**DOI:** 10.3390/healthcare6010014

**Published:** 2018-02-12

**Authors:** Alexandra Trivli, Georgios Dalianis, Chryssa Terzidou

**Affiliations:** Department of Ophthalmology, Konstantopouleio-Patission General Hospital, Agias Olgas 3–5, Nea Ionia, 14233 Athens, Greece; dalianisofth@konstantopouleio.gr (G.D.); cterzidou@yahoo.com (C.T.)

**Keywords:** Conjunctivochalasis, radiofrequencies, dry eye syndrome

## Abstract

The purpose of our study is to present a quick surgical procedure for the treatment of Conjunctivochalasis (CCH) and to evaluate its effectiveness. Thirty consecutive patients, in whom CCH was diagnosed on clinical examination, were investigated for the presence of symptoms of dry eye. The 60 eyes were evaluated according to their symptomatology and the 40 symptomatic eyes were grouped in two stages using the LIPCOF (stage 1, one small fold; stage 2, more than two folds but not higher than the tear meniscus) classification and included in the study. After a subconjunctival injection of lidocaine 20 mg/mL, a medium frequency alternating current (RF) was used, adjusted in low power. With a wide tip, redundant conjunctiva was ablated leaving space between the ablations. Postoperative treatment included eye oint.gentamicin 0.3% with dexamethasone 0.03% three times a day for 5 days. At postoperative day 10, conjunctival edema had subsided and conjunctival epithelium was intact after fluorescein staining. Symptoms had improved in all patients. During follow-up, no complication was detected. Mild conjunctival hyperemia was present in all cases but resolved with standard postoperative medications. To conclude, CCh treatment with RF appears to be a safe, quick, and effective surgical technique. Operation time is less than 10 min and can be performed in an outpatient clinic.

## 1. Introduction

Conjunctivochalasis (CCH) is defined as a redundant, loose, nonedematous inferior bulbar conjunctiva. It is most often located between the globe and the lower eyelid, but not always limited to the inferior bulbar conjunctiva. CCH can be found in the superior and even the whole bulbar conjunctiva [1]. Several theories regarding its etiology have been described, but the exact etiology is still unknown [2,3,4].

CCH is a common cause of ocular surface irritation. It might lead to decreased tear film stability, delayed tear clearance, and therefore an increased concentration of inflammatory markers on the ocular surface and subsequent ocular surface disease. Its clinical significance tends to be overlooked [5,6].

For symptomatic patients, treatment can be either medical or surgical. Medical treatment aims to suppress ocular surface inflammation. Surgical treatment, to remove the redundant conjunctiva, is necessary when the medical approach is unsuccessful. Traditional surgical techniques include excision of the bulbar conjunctiva, suture fixation of the conjunctiva to the sclera, and electrocoagulation of excessive conjunctiva [7,8,9,10]. Amniotic membrane transplantation has been successfully used in the reconstruction of the conjunctival surface after removal of the loose conjunctiva [7,11].

In the framework of this study, we sought to present a new technique to treat CCH, aiming to minimize surgical time without compromising patient outcome.

## 2. Materials and Methods

Thirty consecutive patients (60 eyes) were recruited, while admitting for general ophthalmological examination at our service, when CCH was apparent on clinical examination. Twenty-one patients (40 eyes) were included in the study. Patients’ age ranged between 49 and 85 years (mean 77.1 years). Patients with any kind of anterior surface or lid inflammation as well as obstruction of the lacrimal drainage apparatus were excluded from the protocol. We also excluded patients who had been surgically treated for CCH in the past.

Patients were examined for the presence of CCH symptomatology: epiphora, pain during eye movements or blinking, and common symptoms of dry eye (foreign body sensation, burning, grittiness). 

Upon clinical examination, CCH was evaluated and documented by number of conjunctival folds and position on the lid margin (nasal, medial, temporal). Mechanical lacrimal punctum obstruction due to the nasal folds was evaluated with slit lamp examination, fluorescein staining, fluorescein clearance test (FCT), and the presence of epiphora. In patients with pathological FCT or epiphora, we evaluated the patency of the lacrimal system to rule out any obstruction. Finally, tear break-up time (TBUT) was documented. (Table 1)

For the grading of CCH we used the LIPCOF (stage 1, one small fold; stage 2, more than two folds but not higher than the tear meniscus) classification, dividing CCH into four stages where (0) is no conjunctival fold, (1) is one small fold, (2) denotes more than two folds but not higher than the tear meniscus, and (3) indicates multiple folds higher than the tear meniscus. 

**Figure 1:**
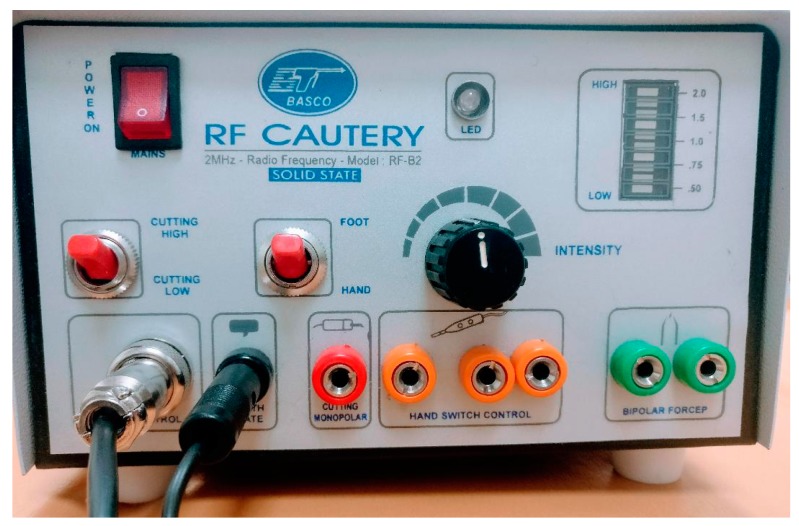
Radiofrequency (RF) cautery equipment used in the procedure.

**Figure 2:**
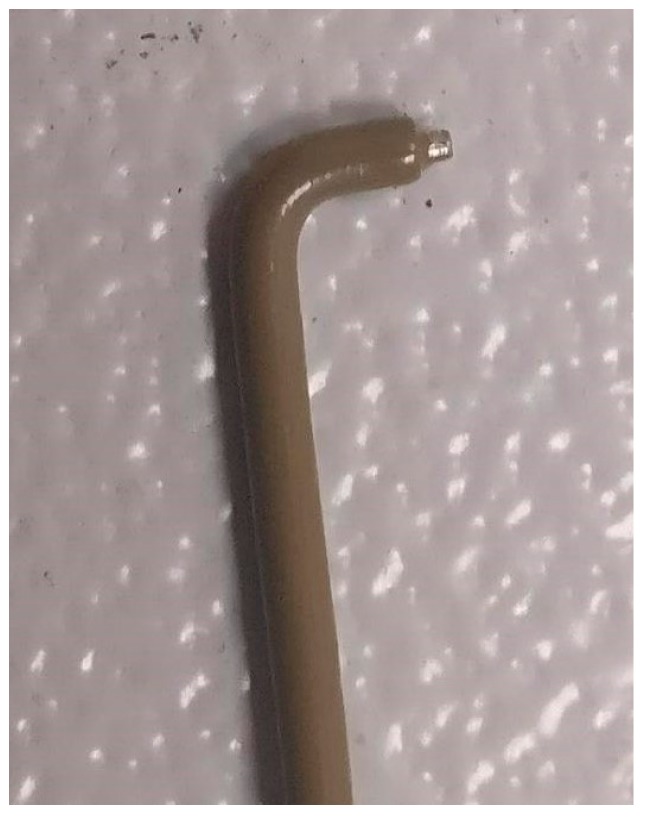
Wide tip used in the procedure.

Topical anesthesia with eye drops of proxymetacaine hydrochloride 5 mg/mL was performed 5 min prior to the procedure. A subconjunctival injection of lidocaine 20 mg/mL was performed with a 25-g needle. A medium frequency alternating current (RF) was used, adjusted in low power (Figure 1). With a wide tip, redundant conjunctiva was ablated, leaving space between the ablations (Figure 2). 

Postoperative treatment included eye oint.gentamicin 0.3% with dexamethasone 0.03% three times a day for 5 days, as well as frequent installation of artificial tears. Prior to the procedure, risks, alternatives, and benefits were explained to the patients and a signed consent form was obtained. 

The Ethics Committee for Human Research of the hospital approved the study. Ethical approval code: 14104/22,05,2017. Date of approval: 22/05/2017. The data were collected by the clinicians who reported the medical records, including surgical procedures and findings. A written informed consent form was obtained by all participants.

## 3. Results

Patients’ age ranged between 49 and 85 years with a mean age of 77.1 years. Follow-up time ranged from 2 to 14 months with a mean of 8 months. 

From the total of 60 eyes examined, atypical symptomatology was present in the 40 eyes included in the study, pain was present in 25 eyes (41.6%), and epiphora was present in 25 eyes (41.6%). 

CCH was evaluated according to the LIPCOF classification system. In this way, 29 eyes were categorized as stage I CCH and 11 eyes were categorized as stage II. (Table 2).

**Figure 3:**
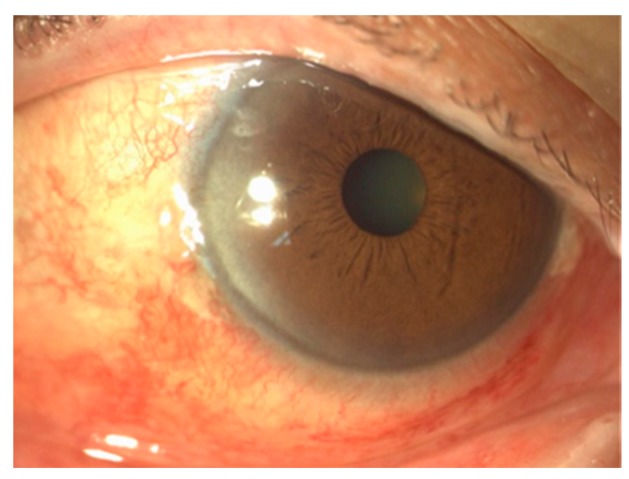
Mild conjunctival hyperemia 24 h postoperative.

**Figure 4:**
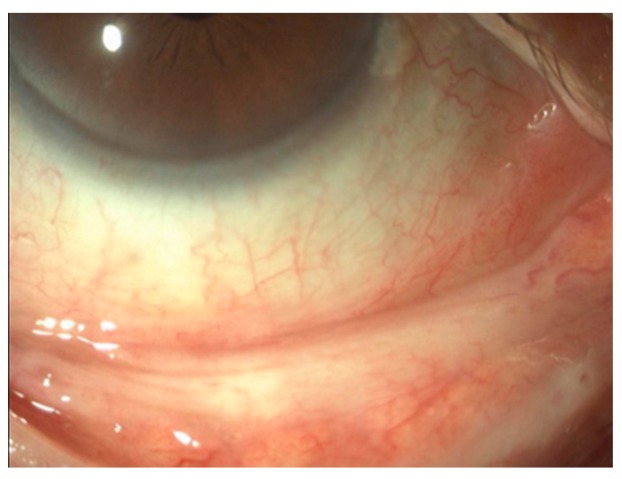
Normal appearance of conjunctiva 10 days postoperative.

At 24 hours postoperative, all eyes showed mild conjunctival hyperemia and edema (Figure 3), which resolved in all of the patients 10 days later with our standard postoperative treatment (Figure 4). 

Symptoms resolved in all patients with stage I CCH and improved significantly for those with stage II CCH regarding duration and intensity. Stage II CCH patients reported that they remained symptom-free for most of the day and that their symptoms were mild and tolerated. All patients reported satisfaction with the result. During follow-up, no major complication was detected.

## 4. Discussion

Conjunctivochalasis is a conjunctival disorder with a poorly understood etiology. Its clinical significance is often underestimated and it can cause various symptoms in patients. These symptoms are associated with Dry Eye Syndrome and can occur mechanically due to the folded conjunctiva. Such symptoms are: foreign body sensation, epiphora, and occlusion of the inferior punctum by conjunctival folds on the margin of the lower eyelid. Ocular surface disease can occur due to the delayed tear clearance [12]. CCH is an increasingly common clinical finding and is often underdiagnosed in older patients.

There are many CCH classification systems proposed in the literature. We used the LIPCOF classification in which CCH is divided into four stages: stage (0), no conjunctival fold; stage (1), one small fold; stage (2), more than two folds but not higher than the tear meniscus; and stage (3), multiple folds higher than the tear meniscus [13].

Various surgical methods have been reported for the treatment of CCH. According to the literature, Hughes was the first to successfully treat this condition by removing a section of the conjunctiva under the lower eyelid and closing the incision with a continuous black silk suture [1]. Furthermore, suture fixation of the conjunctiva to the sclera with 6-0 Vicryl sutures has been described [9]. Additionally, electrocoagulation represents another method, which allows local inflammation to occur and the conjunctiva to attach to the subconjunctival Tenon’s capsule [14,15,16]. 

We previously reported another approach where, after surgical removal of the excess conjunctiva, preserved human amniotic membrane was placed over and sutured with a 10-0 nylon continuous suture to the free conjunctival edges [11]. In the current study, similar to electrocoagulation, our surgical procedure using radiofrequencies achieves conjunctival shortening by burning excessive conjunctiva. The operation is performed in an outpatient clinic setting, is applicable to the conjunctival folds at any location, and patients experience minimal intraoperative and postoperative ocular irritation.

## 5. Conclusions

No recurrence of CCH was noted during our follow-up period. Moreover, surgery usually takes less than 10 min, which is a significant advantage compared to previous procedures reported in the literature.

In conclusion, RF treatment of CCH has a high success rate, a short operation and recovery time, and can be considered a treatment easily accessible for both patients and medical staff.

## Figures and Tables

**Table 1:** Patient’s data documented on clinical examination.

Symptoms	Findings	Tests
Non-typical (itching, burning, foreign body sensation)	CCH severity (number of conjunctival folds)	TBUT
Pain (during blinking or with eye movement)	Epiphora (punctum occlusion by overlapping folds)	FCT
Epiphora or pleolacrimal		

**Table 2:** CCH staging.

No. of Eyes	CCH Stage	Percentage	Pain	Epiphora
0	0	0%	0	0
29	I	72.5%	15	16
11	II	27.5%	10	9
0	III	0%	0	0
40			25	25
